# Immune cell infiltration-associated signature in colon cancer and its prognostic implications

**DOI:** 10.18632/aging.203380

**Published:** 2021-08-04

**Authors:** Dan Deng, Xin Luo, Sifang Zhang, Zhijie Xu

**Affiliations:** 1 Department of Cardiology, Zhuzhou Central Hospital, Zhuzhou 412007, China; 2 Department of Integrated Traditional Chinese and Western Internal Medicine, The Second Xiangya Hospital of Central South University, Changsha 410012, China; 3 Department of Pathology, Xiangya Hospital of Central South University, Changsha 410008, China; 4 National Clinical Research Center for Geriatric Disorders, Xiangya Hospital, Central South University, Changsha 410008, China

**Keywords:** prognosis, colon cancer, immune cell infiltration, tumor mutation burden, immunotherapy

## Abstract

Tumor immune cell infiltration (ICI) has been reported in various studies to be correlated with tumor diagnosis, clinical treatment sensitivity and prognosis. It is an important direction to study the characteristics of immune cell infiltration and develop new prognostic markers to improve the treatment of colon cancer. In this paper, we systematically analyzed the ICI characteristics and obtained three ICI clusters. Then, the ICI scores were constructed and its prognostic implications were discussed. From the results, the ICI score patterns were linked to a great survival difference (p<0.001). A high ICI score was characterized by a higher fraction of plasma cells, CD8^+^ T cells, memory resting CD4^+^ T cells, monocytes, eosinophils and dendritic cells, which had better prognosis. Macrophages and neutrophils were increased in low ICI score patients with decreased overall survival. Immune checkpoint molecules (PDCD1, CD274, LAG3, IDO1, CTLA-4, TIGHT and HAVCR2) were found to be significantly overexpressed in the low ICI score subgroup. In addition, we also studied the correlation between the tumor mutation burden (TMB) and ICI score. This study indicated the ICI score could serve as a potential prognostic biomarker for colon cancer patients’ immunotherapy.

## INTRODUCTION

Colorectal cancer (CRC) is the third most common cancer and accounts for over 8% of all deaths annually worldwide, and colon cancer is its main type [1, 2]. Most colon cancer cases and deaths are attributable to age, physical inactivity, obesity, and genetic and environmental factors [3, 4]. Conventional treatments for colon cancer, including surgery, radiotherapy and chemotherapy, which are invasive treatments, may have a greater impact on patient quality of life [5]. At present, immunotherapy has shown strong antitumor activity in the treatment of many solid tumors, such as melanoma, nonsmall cell lung, renal and prostate cancer [6]. However, neither PD-1/L1 nor CTLA-4 has yet been confirmed to have relevant efficacy in unselected colon cancer, and only the subgroups of deficient mismatch repair (dMMR) or microsatellite instable high (MSI-H) tumors of colon cancer are amenable to checkpoint inhibition [7, 8]. It is necessary to develop new therapeutic markers so that ideal colon cancer subgroups for immunotherapy can be identified.

More and more studies have shown that the TME is connected to the development and metastasis of tumors and significantly affects their diagnosis, survival outcome and clinical treatment sensitivity [9–11]. In-depth study of tumor immune cell infiltration (ICI) and quantification may provide valuable prognostic molecular markers and prognostic models for tumor biology [12]. Naito first described cytotoxic CD8^+^ T cell infiltration in the immune microenvironment of colon cancer as an independent prognostic factor [13]. The prognostic value of cytotoxic and memory T cells in primary colon cancer tumors has been widely reported [14]. Tumor associated macrophages secrete immunosuppressive cytokines, such as interleukin-10 (IL-10) and transforming growth factor- β (TGF- β), can promote the growth and development of tumor [15].

Novel and effective classification methods based on immune infiltrating cells and immune-related genes maybe the key to hierarchical treatment for colon cancer patients [16, 17]. Nowadays, there have been numerous studies searching for prognostic biomarker by integrating multiple elements into a model for prognosis prediction [18]. For example, a prognosis model was conducted based on 12 differential expressed immune genes between healthy and tumor samples [19]. In addition, using Cox analysis, several immune infiltration prognostic signatures based on 22 types of immune cells were build and could serve as important prognostic methods for colon cancer [20–22]. However, the roles of immune infiltration cells and reliable immune-related prognostic signature in colon cancer remain to be fully characterized. In this paper, we systematically analyzed the characteristics of the ICI for colon cancer and used this therapeutic marker to classify and predict the outcome. This marker could be a potential prognostic biomarker and predictive indicator for colon cancer patients’ immunotherapy.

## RESULTS

### Tumor ICI and immunophenotyping

**Figure 1:**
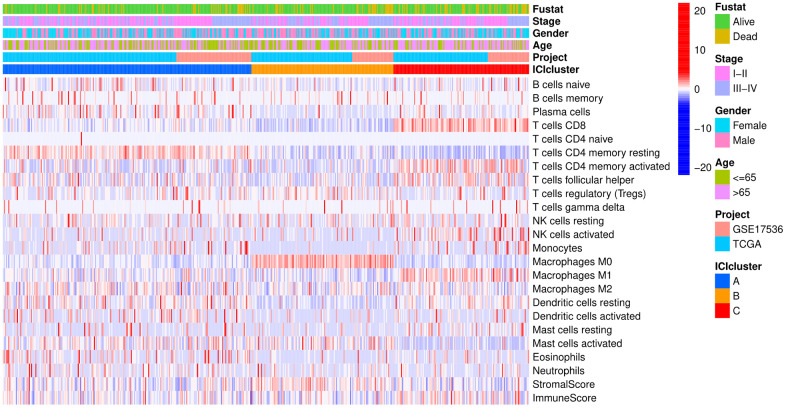
**Heat map.** Rows represent colon cancer samples, columns represent tumor-infiltrating immune cells, red indicates high expression, and blue indicates low expression.

The colon cancer samples were downloaded from TCGA and GSE17536 [23] datasets. In the subsequent analysis, the CIBERSORT and ESTIMATE algorithm were used to quantify the infiltration level of immune cells in colon cancer tissues [24, 25]. The colon cancer patients were divided into three distinct subtypes (ICI clusters A-C) by the K-means algorithm. To analyze the expression of immune cells according to different immune subtypes and clinical characteristics, a heat map of 22 kinds of immune cells associated with different survival statuses, stages, ages and subtypes was drawn (Figure 1).

**Figure 2:**
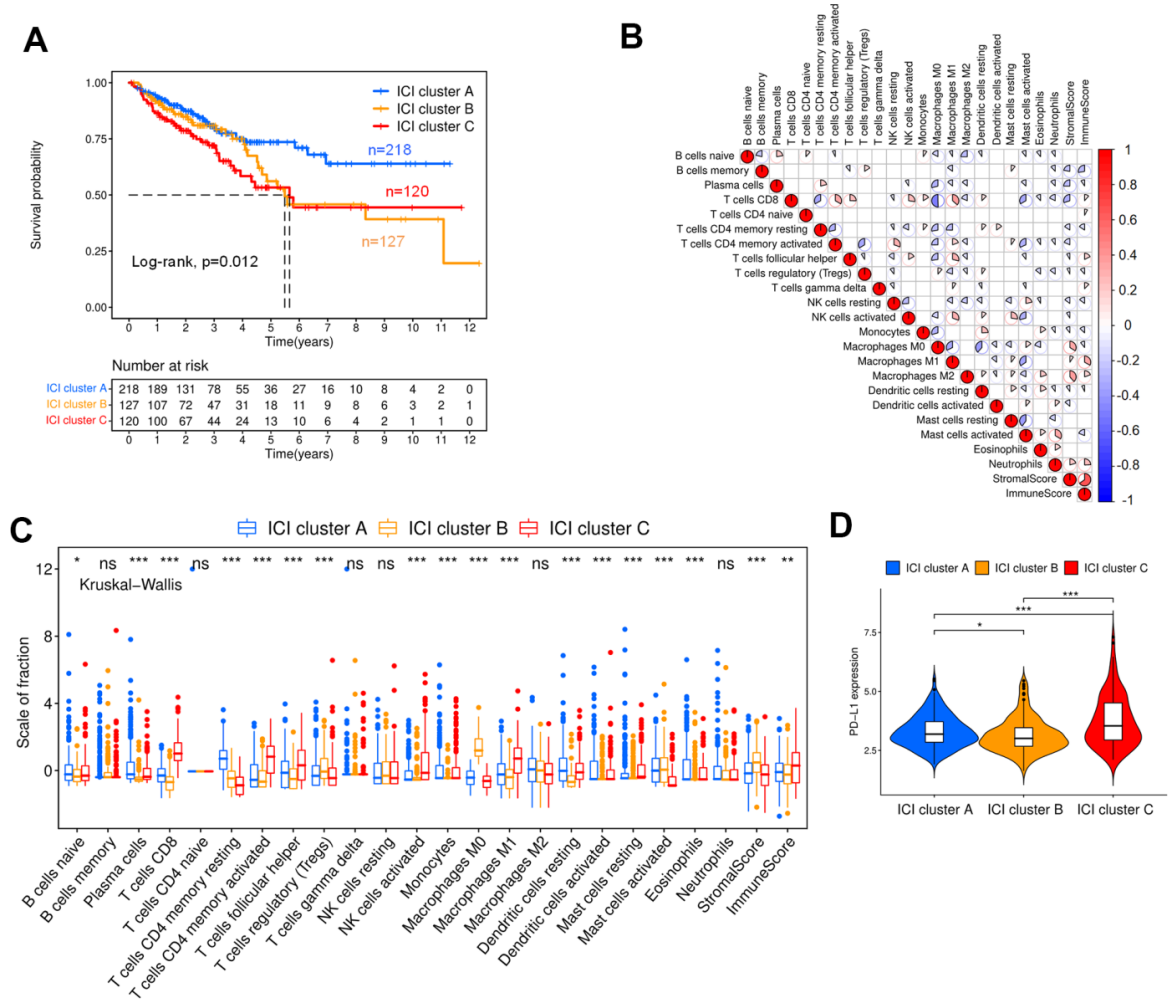
**ICI and immunophenotyping for colon cancer patients.** (**A**) Kaplan-Meier curves for the three ICI clusters (Log-rank test, p=0.012). (**B**) Correlation analysis of tumor-infiltrating immune cells. Blue represents negative correlation, and red represents positive correlation. (**C**) Box plot showing the contents of tumor-infiltrating immune cells within three ICI clusters (Kruskal-Wallis test, ***p< 0.001; **p<0.01; *p<0.05). (**D**) Expression analysis of PD-L1 within three ICI clusters (Kruskal-Wallis test, ***p< 0.001; **p<0.01; *p<0.05).

Figure 2A shows the Kaplan-Meier curve for different ICI clusters, and the patients exhibiting these three ICI clusters show statistically significant survival differences (p=0.012). After that, the correlation between immune cells was analyzed, and the results showed that CD8^+^ T cells had the greatest negative correlation with M0 macrophages and the largest positive correlation with M1 macrophages, the immune scores and stromal scores were negatively correlated with macrophages (Figure 2B). Figure 2C indicates the difference in immune cell contents in ICI clusters of colon cancer patients. ICI cluster A had the best overall survival (OS) and was marked by the highest fraction of plasma cells, memory resting CD4^+^ T cells, dendritic cells, monocytes and eosinophils. ICI cluster B exhibited a significant increase in regulatory T cells and M0 macrophages. In contrast, CD8^+^ T cells, memory-activated CD4^+^ T cells, follicular helper T cells, activated NK cells, M1 macrophages and resting mast cells have the largest proportions in ICI cluster C. Then, we found that the expression level of PD-L1, an important immune checkpoint molecule for colon cancer, presented a significant difference between different ICI clusters, and ICI cluster C was characterized by the highest expression level with poor prognosis (Figure 2D) [26].

### Genotyping

**Figure 3:**
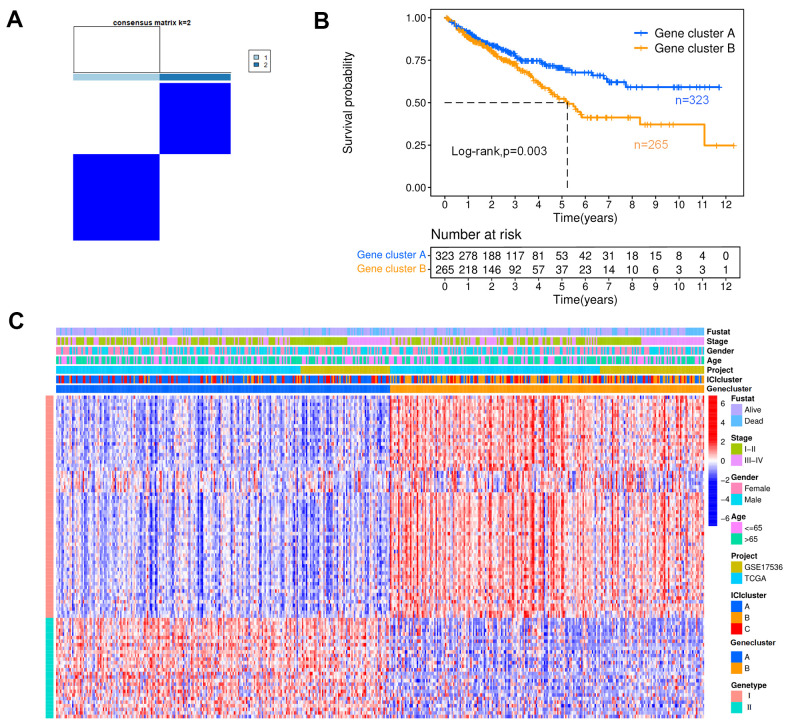
**Genotyping analysis.** (**A**) In a cluster analysis based on the K-means algorithm, the colon cancer patients were classified into two subgroups, designated gene clusters (**A**, **B**). (**B**) The Kaplan-Meier curves for patients with gene clusters (**A**, **B**) (Log-rank test, p=0.003). (**C**) Heat map of DEGs. Rows represent colon cancer samples with different subtypes and clinical status; columns represent tumor-infiltrating immune cells.

To reveal the potential biological characteristics of distinct subtypes, differentially expressed genes (DEGs) were identified among these ICI clusters by the limma package in R software, and 113 DEGs were obtained. The DEGs positively correlated with the gene cluster were marked as type I, and the rest were designated as type II. According to these DEGs, the colon cancer samples were classified into two groups by the K-means algorithm with small intragroup differences and the large differences between distinct subgroups, as shown in Figure 3A; these two groups were named gene cluster A (n=323) and B (n=265). The Kaplan-Meier curve indicated that the patients with the gene cluster A profile had better OS than those with gene cluster B (Figure 3B, p=0.003). Figure 3C shows the heat map of DEGs with different subgroups and clinical statuses.

### ICI score prognostic signature

In this section, 90 characteristic genes were screened from the DEGs by the Boruta package in R software (Supplementary Table 1). After that, the scores of type I (n=62) and type II (n=28) characteristic genes were calculated by principal component analysis (PCA), named *S*_PCA I_ and *S*_PCA II_, respectively. The ICI score of each colon cancer patient could be obtained as follows:

$$\text{ICI score=}S_{\text{PCA}\;\text{I}}-S_{\text{PCA}\;\text{I}}$$

**Figure 4:**
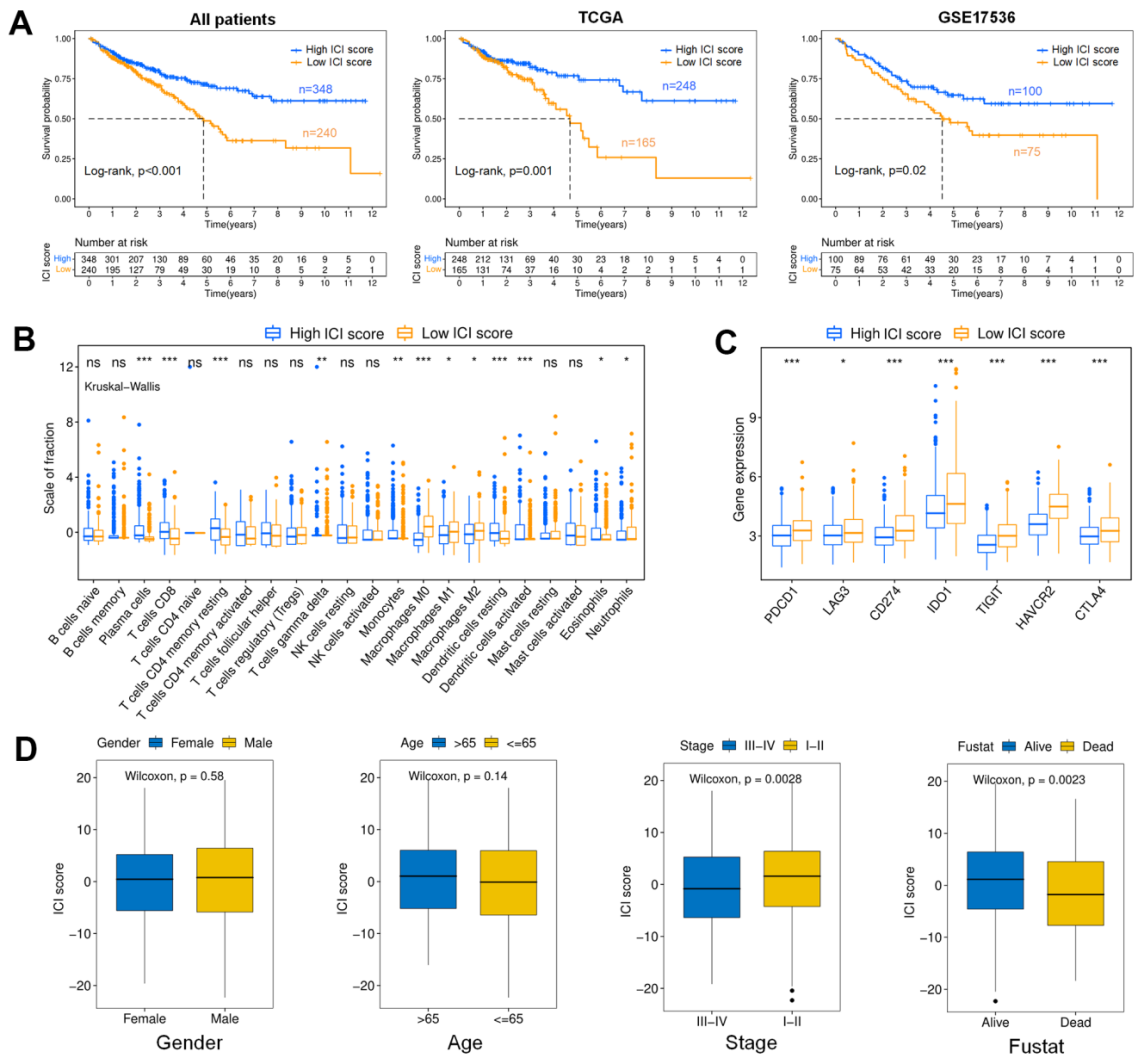
**Analysis of the ICI score.** (**A**) Kaplan-Meier curves for colon cancer patients with high and low ICI scores (Log-rank test). All patients (p<0.001); TCGA (p=0.001); GSE17536 (p=0.02). (**B**) Boxplot showing the contents of tumor-infiltrating immune cells within the high and low ICI score subgroup (Kruskal-Wallis test, ***p< 0.001, **p<0.01, *p<0.05). (**C**) immune checkpoint gene expression analysis for colon cancer in high and low ICI score subgroups. (Kruskal-Wallis test, ***p<0.001, **p<0.01, *p<0.05). (**D**) Boxplots showing the difference of ICI scores with different Clinical characteristics (Wilcoxon test). Gender (p=0.58); Age (p=0.14); Stage (p=0.0028); Fustat (p=0.0023).

The colon cancer patients could be divided into high (n=348) and low (n=240) ICI score subgroups with the optimal cutoff value. The survival rate of the high ICI score subgroup was significantly higher than that of the low ICI score subgroup at the same time point, suggesting that colon cancer patients with high ICI scores had a better prognosis, as shown in Figure 4A (p<0.001). Moreover, it could be found that there was a significant difference in survival between high and low ICI score patients in TCGA (p=0.001) and GSE17536 (p=0.02), respectively. The high ICI score subtype was marked by a high proportion of plasma cells, CD8^+^ T cells, memory resting CD4^+^ T cells, monocytes, dendritic cells and eosinophils. In the low ICI score subtype, macrophages and neutrophils contributed a relatively greater fraction. (Figure 4B). We further analyzed the immune activity of the immune checkpoint relevant molecules in high and low ICI score subgroups (*LAG3, PDCD1/PD-1, IDO1, CD274/PD-L1, CTLA-4, TIGHT, HAVCR2*) [27, 28]. We found that all immune checkpoint molecules were significantly overexpressed in the low ICI score subgroup, which had poor outcomes (Figure 4C). The ICI score showed statistical differences between alive and dead (p=0.0023), stage I-II and III-IV (p=0.0028) patients, in which surviving and stage I-II patients associated to higher ICI scores, however, there was no obviously difference in gender and age (Figure 4D).

**Figure 5:**
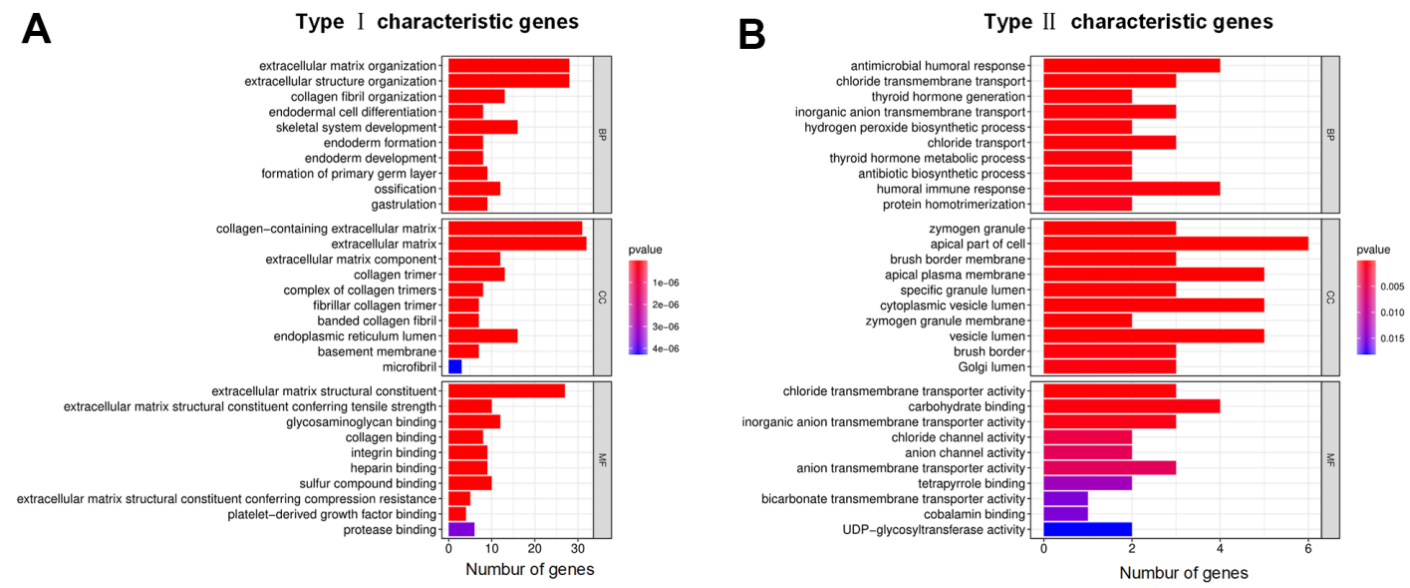
**GO annotations for characteristic DEGs.** (**A**) Bar chart for type I characteristic genes. (**B**) Bar chart for type II characteristic genes. The abscissa represents the number of characteristic genes, and the ordinate shows the GO term. BP represents biological process, CC represents cell component, and MF represents molecular function.

**Figure 6:**
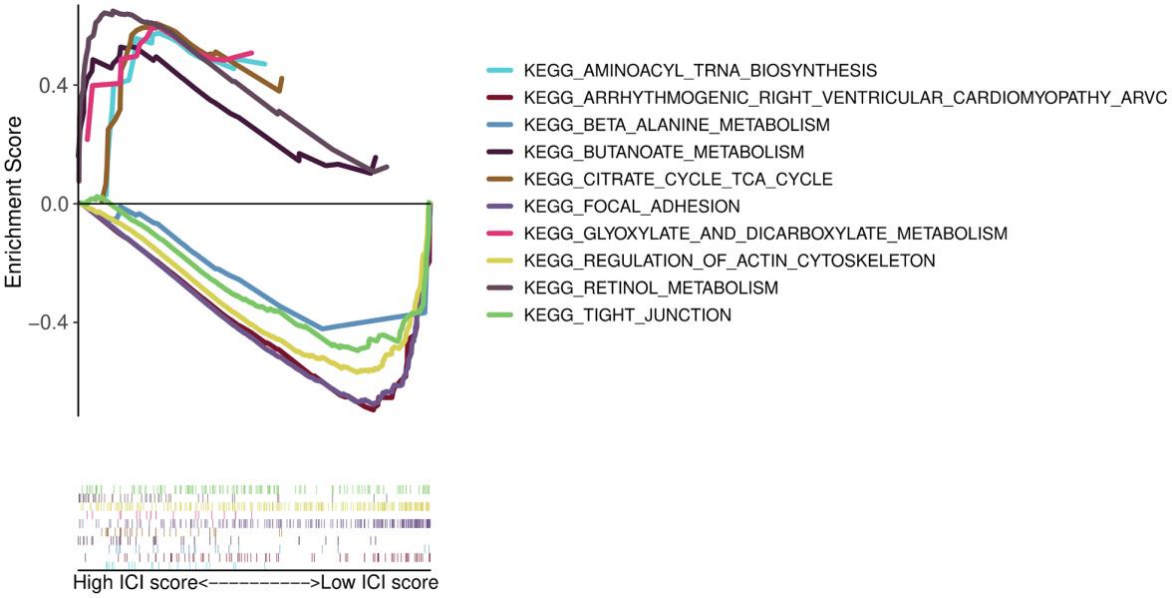
GSEA enrichment plots.

Gene Ontology (GO) enrichment analysis was used to define the different biological functions in different DEG types. The results showed that type I characteristic DEGs were mainly enriched in extracellular matrix, extracellular structure organization, collagen-containing extracellular matrix and extracellular matrix structural constituent (Figure 5A). Type II characteristic DEGs were primarily enriched in antimicrobial humoral response, humoral immune response, apical part of cell and carbohydrate binding (Figure 5B). The results of GSEA showed that aminoacyl tRNA biosynthesis, glyoxylate and dicarboxylate metabolism, butanoate metabolism, citrate cycle/TCA cycle, and retinol metabolism were enriched in the high ICI score subgroup, whereas beta alanine metabolism, tight junction, regulation of actin cytoskeleton, arrhythmogenic right ventricular cardiomyopathy (ARVC), and focal adhesion were enriched in the low ICI score subgroup (Figure 6).

### Association analysis of the TMB and ICI score

**Figure 7:**
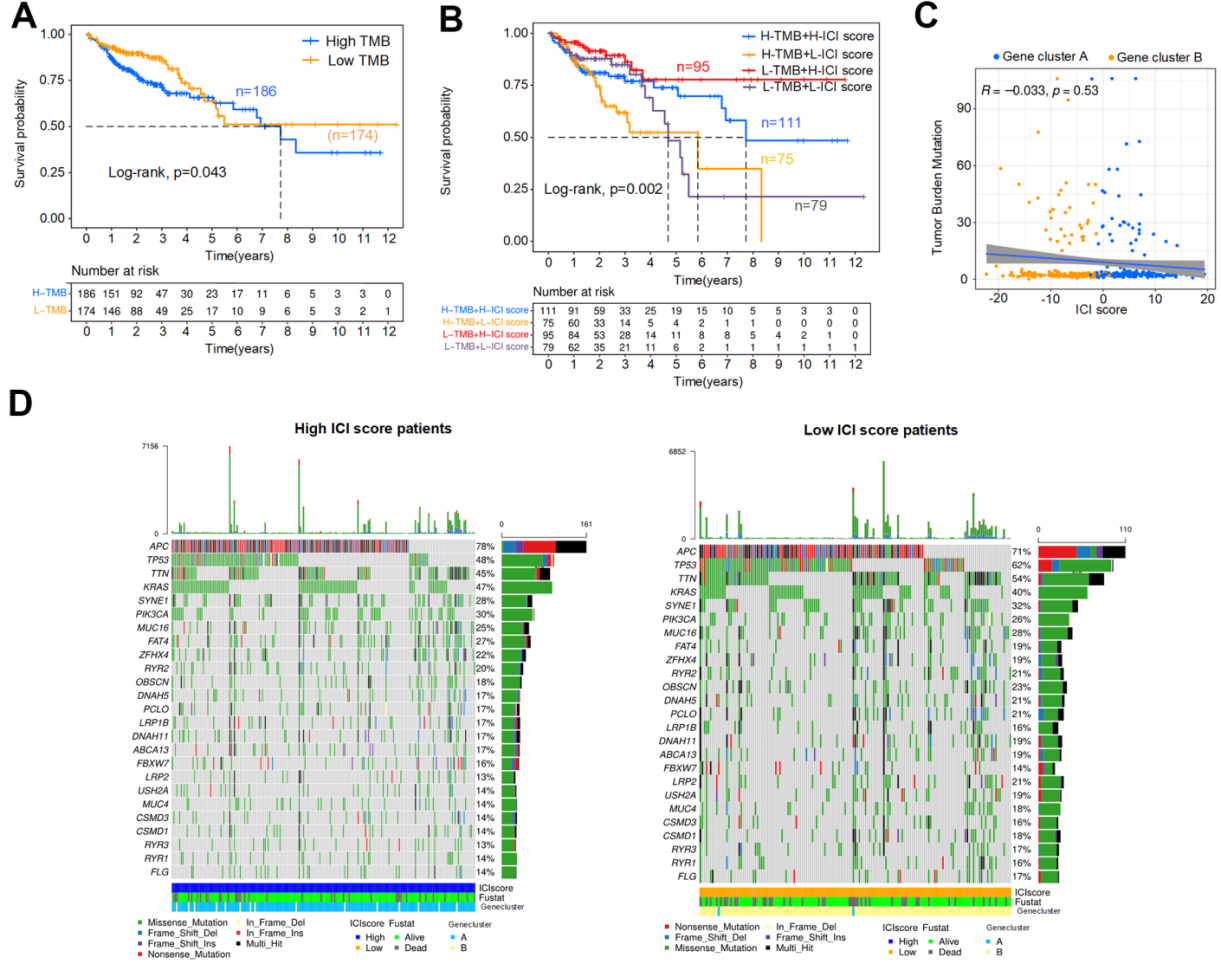
**Association analysis of the ICI score and TMB.** (**A**) Kaplan-Meier curve for high and low TMB colon cancer patients (Log-rank test, p=0.043). (**B**) Kaplan-Meier curve for combined subgroups of the ICI score and TMB (Log-rank test, p=0.002). (**C**) Correlation analysis between the TMB and ICI score, Spearman correlation coefficient = -0.033, p=0.53. (**D**) Waterfalls plot of driver gene mutations in the high and low ICI score subtypes. Rows represent colon cancer samples with different subgroups, columns represent genes, and the mutation type is indicated by different colors.

This section analyzed the internal relationship between ICI score and tumor mutation burden (TMB), considering TMB is a potential predictor of response to immune checkpoint inhibitors in MSI-H colon cancer [29]. First, we downloaded the TMB data of colon cancer patients from TCGA database and classified these patients into high (n=186) and low (n=174) TMB subtypes. As shown in Figure 7A, the OS of the low TMB group was better (p=0.043). The Kaplan-Meier curve showed significant differences in OS among the combined subgroups (H-TMB+H-ICI score, H-TMB+L-ICI score, L-TMB+H-ICI score and L-TMB+L-ICI score), and the ICI score subgroups showed significant survival differences in both the high and low TMB subtypes (Figure 7B, p=0.002). Among them, the L-TMB+H-ICI subgroup patients had the best prognosis. To explore the intrinsic relationship between the TMB and ICI scores, difference and correlation analyses were conducted between TMB and ICI scores. The correlation analysis confirmed that the ICI score was not obviously correlated with the TMB, as shown in Figure 7C. Further, the mutation annotation files of TCGA cohort between the high and low ICI score subgroups were analyzed, and the top 25 driver genes with the highest alteration frequency are shown in Figure 7D. These analyses revealed that 8 driver genes (*APC, TTN, TP53, KRAS, LRP2, FAT4, OBSCN, LRP2*) were clearly different in alteration frequency (the value of difference >= 7%) between the two ICI score subgroups, but only TP53 showed a statistically significant difference (Table 1).

**Table 1:** The top 20 mutation genes were clearly different in alteration frequency between the two ICI score subgroups in colon cancer patients.

**Gene**	**H-mutation (%)**	**L-mutation (%)**	**p-value**
TP53	99(48%)	95(62%)	0.0139
SDK1	19(9%)	27(18%)	0.0295
RNF43	16(8%)	24(16%)	0.0303
DNAH7	12(6%)	25(16%)	0.0023
DSCAM	14(7%)	21(14%)	0.0469
PTPRT	13(6%)	21(14%)	0.0301
PDZD2	12(6%)	21(14%)	0.0184
DOCK2	11(5%)	19(12%)	0.0290
NIPBL	11(5%)	19(12%)	0.0290
PKD1L1	9(4%)	18(12%)	0.0161
ZNF423	10(5%)	17(11%)	0.0453
NBEAL2	8(4%)	18(12%)	0.0086
GPR158	9(4%)	17(11%)	0.0269
TRIOBP	8(4%)	17(11%)	0.0150
PKDREJ	9(4%)	16(10%)	0.0440
MKI67	9(4%)	16(10%)	0.0440
ERICH3	9(4%)	16(10%)	0.0440
SIPA1L1	8(4%)	16(10%)	0.0254
SHPRH	8(4%)	16(10%)	0.0254
CDH18	7(4%)	16(10%)	0.0137

## DISCUSSION

Increasing evidence shows that the TME is closely related to the tumor growth, invasion and metastasis of colon cancer [30, 31]. At present, the construction of these immune infiltration prognostic signatures for colon cancer mainly focuses on the identification prognosis-related molecules by Cox regression analysis [19–21]. ICI scores are obtained from unsupervised clustering and PCA algorithm between patients with different ICI landscape. Moreover, its prognostic value for immunotherapy have been discussed in head and neck squamous cell carcinoma [32]. However, the detailed functions and features of ICI scores have not been elucidated in colon cancer.

From the immunophenotyping results, the ICI cluster A subgroup had the best prognosis, with the highest proportions of plasma cells, memory resting CD4^+^ T cells, dendritic cells, monocytes and eosinophils. However, increased infiltration of CD8^+^ T cells, memory-activated CD4^+^ T cells, follicular helper T cells, activated NK cells, M1 macrophages and resting mast cells were correlated with decreased OS. To quantify the ICI patterns of individual tumors in colon cancer, the ICI score based on immune-related genes and tumor ICI was used to predict prognosis, and it showed the best survival differences compared with immunophenotyping and genotyping. We found plasma cells, CD8^+^ T cells, memory resting CD4^+^ T cells, monocytes and dendritic cells exhibited relatively higher infiltration levels in the high ICI score patients with increased OS. In the past research, CD4^+^ and CD8^+^ T cells have been well documented to play important roles in antitumor immune responses and as clinically useful prognostic markers in colon cancer [33–35], dendritic cells can present tumor antigens and activate the naive T cells [36], and the increase of plasma cells indicates an obvious humoral immune response [37]. These results indicating an obvious an antitumor immune response in the high ICI score subgroup. At the same time, CD8 ^+^ T cells and M0 macrophages showed a large negative correlation in the correlation analysis of tumor infiltrating cells. These results suggest that the low level of ICI and the increased expression of tumor-associated macrophages may contribute to the poor prognosis of patients with colon cancer. From the GO enrichment analysis, type I characteristic DEGs were mainly distributed in the functions related to the extracellular matrix and type II characteristic DEGs were mainly enriched in antimicrobial humoral response, humoral immune response and apical part of cell.

Immunotherapy has become a major effective treatment modality for multiple types of solid cancers [38, 39]. This study analyzed the expression of PD-L1 in different ICI clusters and found that the highest expression of PD-L1 in ICI cluster C patients with poor prognosis, and PD-1/L1 has been identified as a possible target for immunotherapy in MSI-H/dMMR colon cancer [20, 40]. In addition, potential and known immune checkpoint molecules (PDCD1, CD274, LAG3, IDO1, CTLA-4, TIGHT and HAVCR2) were found to be significantly overexpressed in the low ICI score subgroup associated with poor outcomes. The overexpression of immune checkpoint molecules on immune cells will inhibit the function of immune cells, which causes the body to be unable to produce an effective antitumor immune response, and suppress the immune function of low ICI score patients [41, 42]. For example, the high expression of PD-L1 in tumor tissue inhibits the function of tumor infiltrating CD8 ^+^ T cells [43], and the CTLA-4 will exert its inhibitory functions on the initial T cells activation, leading to tumor immune escape [44].

High TMB is generally associated with poor OS [45], this is consistent with our research result, although the survival difference between the high and low TMB colon cancer patients was not remarkable. It is worth noting higher TMB (highest 20% in each histology and the cut-point was 52.2MB) is associated with better OS for immune checkpoint inhibitor treatments of colon cancer [46]. These may be related to the overall low TMB in our samples. In the association analysis of ICI score and TMB, the ICI score subgroups showed notable survival differences within both high and low TMB subtypes, and the L-TMB+H-ICI score subgroup patients had the best OS. Multiple genes, including *TP53*, one of the most common type of mutations in colon cancer, showed significant differences in mutation frequency between patients with high and low ICI scores. In the previous studies, *TP53* mutation has been confirmed to be related to immune response and progression of colon cancer [47, 48].

Our study results implied that a high ICI score was characterized by a higher fraction of plasma cells, CD8+ T cells, memory resting CD4+ T cells, monocytes, eosinophils and dendritic cells with increased OS. And the low level of T cells infiltration, overexpressed macrophages, neutrophils and multiple immune checkpoint molecules may lead to poor prognosis in colon cancer. In summary, the ICI score could be a potential prognostic biomarker for colon cancer patients’ immunotherapy. However, the findings demand further evaluated by a larger cohort of samples and clinical trial.

## MATERIALS AND METHODS

### Colon cancer data and samples

This paper was conducted in R 4.0.3 software (https://www.r-project.org/). The level-three transcriptome RNA-sequencing data (HTSeq-FPKM) and clinical data of 473 primary colon cancer samples were derived from TCGA database (https://portal.gdc.cancer.gov/). At the same time, 177 colon cancer samples were downloaded from the GEO (GSE17536, https://www.ncbi.nlm.nih.gov/geo/) database. After that, the expression data downloaded from these two databases were sorted and annotated with Perl software. To eliminate the differences in the data measured in these two databases, the FPKM value expressed in TCGA database was transformed into transcripts per kilobase million (TPM) using the limma package in R. Then, the sample expression data matrixes from TCGA and the GEO database were combined for subsequent analysis.

### CIBERSORT and ESTIMATE algorithm

In this section, the CIBERSORT package of R software was used to quantify the infiltration level of immune cells in colon cancer patients. The number of simulations was set as perm=1000, and the screening criterion was a p-value<0.05. The immune and stromal score and their comprehensive score were evaluated by ESTIMATE for each colon cancer sample.

### Immunophenotyping and genotyping

The ConsensClusterPlus package of R software and the K-means algorithm were used to classify colon cancer patients, and the maximum K was set to 9 to select the optimal classification. The classification of colon cancer patients mainly includes immunophenotyping and genotyping, and the subgroups were called ICI clusters and gene clusters, respectively. ICI cluster classification was based on the content of immune cells, and gene clusters were based on the expression of DEGs.

### The DEGs identified and ICI score signature constructed

The limma package was used to identify DEGs between different ICI clusters with the following criteria: |log_2_ FC|>1 and FDR<0.05. According to the expression of DEGs in gene clusters, DEGs were divided into two types. The DEGs were marked as type I if their expression was positively correlated with genotyping, and the rest were marked as type II. The Boruta package was used to find the characteristic genes. Then, using the PCA algorithm to calculate the score of characteristic genes for type I and type II, marked *S*_PCA I_ and *S*_PCA II_, respectively. The ICI score of each colon cancer patient could be calculated as:

$$\text{ICI score=}S_{\text{PCA}\;\text{I}}-S_{\text{PCA}\;\text{II}}$$

Then, the surv_cutpoint function was used to analyze the ICI score of patients and find the optimal cutoff point. The colon cancer patients were divided into high and low ICI score subgroups based on this cutoff point.

### Survival analysis and immunocyte difference analysis

To study whether the different subgroups were related to the prognosis in colon cancer patients, Kaplan-Meier survival curves of different subgroups were drawn, and the log rank test was used to evaluate whether the differences were statistically significant. All Kaplan-Meier survival analysis excluded patients whose survival time was less than 30 days. Moreover, the differences in fractions of tumor infiltrating immune cells in different clusters were analyzed in this paper by Kruskal-Wallis test.

### GO function enrichment analysis and GSEA

GO functional analysis was carried out to explore the biological functions of ICI DEGs for colon cancer, and bar plots for type I and type II characteristic genes were drawn. The Gene Set Enrichment Analysis (GSEA) of high and low ICI score subgroups was conducted by GSEAv4.1 software (http://www.gsea-msigdb.org/gsea/index.jsp), and the number of permutations was set to 1000.

### Association analysis of the ICI score and TMD

The mutation data for the colon cancer patients were downloaded from TCGA database. The TMB was analyzed by counting the total number of nonsynonymous mutations in the colon cancer sample. Then, four subtypes were obtained based on the combination of the TMB the ICI score: H-TMB+H-ICI score, H-TMB+L-ICI score, L-TMB+H-ICI score and L-TMB+L-ICI score. Using maftools package of R software to identify the driver genes, and the top 25 driver genes with the highest alteration frequency were shown in waterfall plots and top 20 mutation genes with statistical difference were listed.

## Supplementary Material

Supplementary Table 1
