# Supplementary material for: Immune cell infiltration-associated signature in colon cancer and its prognostic implications
Source: Aging (Albany NY). 2021 Aug 4;13(15):19696–709. doi: 10.18632/aging.203380 (PMC8386549; doi:10.18632/aging.203380)
Supplement: Supplementary Table 1 [file aging-13-203380-s001.pdf]

SUPPLEMENTARY TABLE

Supplementary Table 1. The 90 characteristic genes were screened by the Boruta package in R software.

Type	Gene
I (n=62)	MMP9, ITGBL1, THBS2, COMP, BGN, GAS1, SFRP2, FNDC1, CYP1B1, COL10A1, SFRP4, MARCO, COL11A1, SPP1, FN1, AEBP1, ASPN, PRELP, COL1A1, THBS4, DES, NKG7, CCL5, IDO1, CXCL9, CXCL10, GZMA, CDH11, LTBP1, DPYSL3, MSRB3, ANTXR1, COL8A1, FIBIN, OLFML2B, COL8A2, VCAN, COL12A1, MATN3, FBN1, COL5A2, LRRC15, INHBA, COL5A1, FAP, MFAP5, MXRA5, SULF1, TAGLN, COL3A1, PLN, LUM, COL1A2, MGP, MMP11, SPOCK1, OLR1, GBP4, POSTN, CTHRC1, CNN1, MYH11
II (n=28)	CLCA1, ITLN1, FCGBP, SPINK4, MUC2, CLCA4, REG4, ZG16, B3GNT6, PIGR, HEPACAM2, DUOXA2, LCN2, DUOX2, ATOH1, CA4, IGLJ3, CEACAM7, UGT2B17, VSIG2, MS4A12, NXPE4, TCN1, REG1A, OLFM4, REG3A, DEFA5, SLC26A3
